# Concurrence of villous adenoma and non-muscle invasive bladder cancer arising in the bladder: a case report and review of the literature

**DOI:** 10.1186/1471-2490-13-36

**Published:** 2013-07-20

**Authors:** Yoichiro Kato, Susumu Konari, Wataru Obara, Tamotsu Sugai, Tomoaki Fujioka

**Affiliations:** 1 Department of Urology, Iwate Medical University, Uchimaru, 020-8505, Moriokashi, Iwate, Japan; 2 Department of Urology, Ninohe prefectural hospital, Ninohe, Iwate, Japan; 3 Department of Diagnostic Pathology, Iwate Medical University, Morioka, Iwate, Japan

**Keywords:** Urinary bladder neoplasms, Villous adenoma, Urothelial carcinoma

## Abstract

**Background:**

Villous adenoma arising in the urinary tract is rare tumor. Most cases have been identified as benign neoplasm in the colon. Villous adenoma of the gastrointestinal tract is thought arise from premalignant polyps. Here, we report a case of concurrence of villous adenoma and non-muscle invasive bladder cancer.

**Case presentation:**

An 85-year-old woman presented at our office because of gross hematuria. Cystoscopic examination detected two papillary tumors in the bladder. Each tumor was resected and diagnosed, respectively. Histopathology confirmed that the resected one tumor was a villous adenoma, and the other was urothelial carcinoma (T1, high grade). Immunostaining for cytokeratin (CK) 7, CK20 and Ki-67 confirmed that CK7: (−), CK20: (+) and Ki-67: (<=30%) in villous adenoma while CK7: (+), CK20: (+), and Ki-67: (70%) in urothelial carcinoma. Three months later from TUR, urothelial carcinoma recurred in the trigone. She received adjuvant intravesical immunotherapy with BCG post TUR for the recurrence site.

**Conclusion:**

There were no specific findings on ultrasonography, CT, MRI or cystoscopic examination morphologically. Therefore, pre-pathological villous adenoma of the bladder is extremely difficult to diagnose. There are some case reports of solitary villous adenoma in the bladder or with coexisting adeno carcinoma. However, to the best of our knowledge, this is only the second report of villous adenoma in the bladder of coexisting urothelial carcinoma that has been published in the literature. Premalignant villous adenoma of the bladder is extremely rare and difficult to diagnose without histologic examination. Any suspicious lesion of the bladder should be biopsied and/or resected to confirm histology.

## Background

Villous adenoma first reported by Norbury LE in 1928 [1] is now recognized as a premalignant polyp of the gastrointestinal tract. Up to two-thirds of the lesion occurs in the rectum. There are no differences in distribution between men and women and a peak incidence in the 60’s and 70’s [2]. Whenever possible, local excision and sphincter preservation is the procedure of choice for accessible lesions with favorable characteristics. However, the recurrence is seen in up to 40% of cases even despite complete excision in the rectal [3].

On the other hand, the villous adenoma in the urinary tract is rare. The most common coexisting tumor is adenocarcinoma which is associated with urachus tumors [4]. Typical clinical presentations are hematuria and irritative symptoms [5]. The prognosis of pure villous adenoma in the urinary tract is excellent.

## Case presentation

An 85-year-old woman with no significant past medical history, including colon cancer but urethral caruncle two years ago presented at our office because of painless gross hematuria.

Physical examination showed no remarkable findings. Laboratory abnormalities were no pyuria but microscopic hematuria and mild anemia.

**Figure 1:**
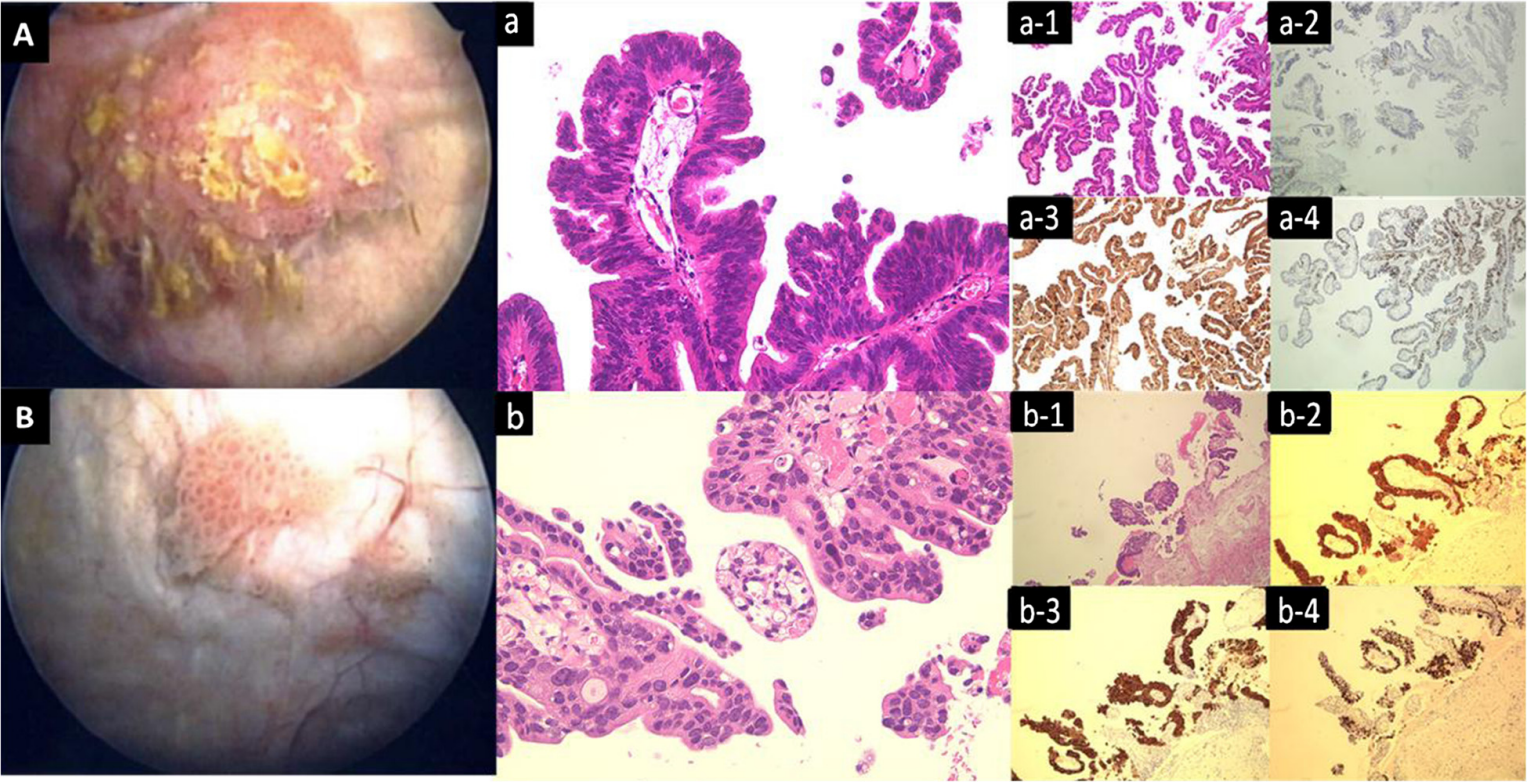
**Cystscopy findings and HE histopathologic findings and representative immunohistochemical findings for cytokeratin (CK) 7, CK20 and Ki-67. A** and **a**, Villous adenoma: The papillary tumor with peduncle was in the right wall. **B** and **b**, Urothelial carcinoma: The papillary tumor in the left wall in this case the columnar epitherium of tumor was longer than that of the villous adenoma. **1**, HE. **2**, CK7. **3**, CK20. **4**, Ki-67.

Initial ultrasonography examination (US) revealed a mass, 15 mm in diameter, on the right bladder wall. Subsequent cystoscopic examination detected two papillary tumors in the bladder. This confirmed that the bigger one was same size as that on US on the right upper wall with a peduncle. The second was 9 mm with some micro satellite tumors on the left wall. Both of them were typical papillary tumors morphologically (Figure 1A, B).

An enhanced computed tomographic (CT) scan of the bladder showed two masses. Larger one measuring 16 mm was in the right wall and the other was 9 mm mass in the left bladder wall, with enhancement. Moreover, no other masses or no enlarged lymph nodes were seen except for the bladder.

On magnetic resonance imaging (MRI), T2-weighted images revealed a solid, low intensity mass measuring 15 mm and the mass could be limited to the right bladder wall without muscle invasion. No urachal remnant was confirmed in a sagittal section. For the 9 mm mass, MRI revealed that it was limited to the left bladder wall and was also without muscle invasion.

At this point, we could diagnose multiple non-muscle invasive bladder cancer. Cold punch biopsy was performed at cystoscopic examination. We performed a biopsy only in the right side tumor. The histology confirmed it to be a villous adenoma. According to this result, a colonoscopy examination was then performed and no neoplastic lesion was found. TUR was performed after preoperative examinations. Each tumor was resected and diagnosed, respectively.

Histopathology confirmed that the resected tumor on the right wall was a villous adenoma, and the left one was urothelial carcinoma (T1, high grade). Histologically, the former consisted of tall columnar epithelium which formed a villous pattern with vessels and stroma consisting of hypo connective tissues, few nuclear atypia, and few mitotic figures. The urothelial tumor consisted predominantly of papillary growth which formed the structure of the gland (Figure 1a, b). Immunostaining for cytokeratin (CK) 7, CK20 and Ki-67 confirmed that in villous adenoma, for CK20 the staining was positive, Ki-67 was less than 30%, while CK7 was negative. In distinct contrast, there was very strong positive staining for both cytokeratins moreover, the staining for Ki-67 was quite strong at approximately 70% in urothelial carcinoma.

These findings supported a diagnosis of villous adenoma and urothelial carcinoma arising in the bladder. On first follow up on the cystoscopic examination, which was held 3 months later, small papillary tumors were found in the trigone. Based on this finding, TUR was performed for the recurring lesion. The recurring tumor was diagnosed as urothelial carcinoma pathologically. She received adjuvant intravesical immunotherapy with BCG. After BCG therapy, she has been followed by cystoscopic examination for every three months. At the last follow-up, 24 months after surgery, no local recurrences were detected.

## Discussion

The clinical manifestations included gross hematuria, but none led to an increased suspicion of villous adenoma. There were no specific findings on ultrasonography, CT, MRI or cystoscopic examination morphologically. Therefore, pre-pathological villous adenoma of the bladder is extremely difficult to diagnose.

We have summarized several isolated case reports and two case series of villous adenoma with and without other types of carcinoma in Table 1[5-24]. Our case in which the villous adenoma coexists with urothelial carcinoma is the second case report in the table.

**Table 1:** Summary of villous adenoma of the urinary tract with and without other types carcinoma

	**VA**	**VA + AD**	**VA + AD + UC**	**VA + UC**
**1976-2009 isolated reports**[6-23]	**13**	**7**		
**Cheng et al.**[5]	**15**	**8**		
**Seibel et al.**[24]	**6**	**9**	**2**	**1**
**Our case**				**1**
**Total**	**34**	**24**	**2**	**2**

Moreover, clinical features of published reports according to the types of concurrence carcinomas are presented in Table 2[5-24]. Though there are some variabilities of fineness, this table shows us some differences and similarities between solitary villous adenoma and concurrence villous adenoma and adenocarcinoma. The other types including our case are too small number of cases to compare. Age, gender, predominant symptoms and locations are similar between solitary villous adenoma and concurrence villous adenoma and adenocarcinoma. On the other hand, treatments and recurrence and progression rate are differences between groups. Though, the cases of concurrence villous adenoma and adenocarcinoma were received more radical therapies than solitary villous adenomas, the rate of the recurrence or progress were higher than solitary villous adenomas. These outcomes would be attributed to the malignant grade of adenocarcinoma.

**Table 2:** Summary of clinical features of published reports according to the types of concurrence carcinomas*

**Primary neoplasm (n)**	**Age(range/ median)**	**Gender (M/F)**	The top three symptoms (%)***	**The top three locations (%)**	**The top three treatments (%)**	**Recurrence or progress rate % (n)**
VA (34)	(23–88 /61.5)	(19/14)**	1. Hematuria (32.4)	1. Bladder dome (26.5)	1. TUR (32.4)	2.9%: (1/34 cases)
			2. Irritative symptom (17.6)	2. Bladder (20.6)	2. Unknown (20.6)	
			3. Unknown (17.6)	3. Posterior wall (8.8)	3. None (17.6)	
VA + AD (24)	(31–94 /65.5)	(14/10)	1. Hematuria (33.3)	1. Bladder (37.5)	1. TUR (29.2)	20.8%: (5/24 cases)
			2. Irritative symptom (29.2)	2. Urethra (33.3)	2. Total cystectomy (20.8)	
			3. Abdminal pain (8.3)	3. Bladder dome (16.7)	3. Partial cystectomy (16.7)	
VA + AD + UC (2)	(76–92 /84)	(2/0)	1. Unknown (100)	1. Bladder (100)	1. TUR (100)	0%: (0/2 cases)
VA + UC (2)	(66–85 /75.5)	(1/1)	1. Hematuria (50.0)	1. Bladder (33.3)	1. Partial cystectomy (50.0)	100%: (2/2 cases)
				1. Urachus (33.3)		
					1. TUR (50.0)	
				1. Lateral wall (33.3)		

*Including overlap feautures (some indexes > =100%). **No description about sex in one case report. ***No description about symptoms in one case series.

There is no certain reason why the glandular lesion was in the urinary tract. However, in the 5th fetal month the end of Wolffian duct opens into the cloaca which is cavity of area around the anus. By the end of 7th fetal month, the cloaca is divided by the urorectal septum into a dorsal rectum and a ventral urogenital sinus. The villous adenoma could originate from the rest of cloacal epithelium [4,6]. Moreover, there were three case reports of villous adenoma arising after colocystoplasty [25]. That is glandular lesions coming from urine invasion would be one of the factors to generate neoplasms.

In the present case, there were clear differences in the histological findings between the villous adenoma and the urothelial carcinoma as indicated in the ‘Case report’ (Figure 1a, b).

Immunohistochemical studies demonstrated diagnostic features indicating positive staining for CK20 but negative for CK7 and focally positive for Ki-67 in villous adenoma. In contrast, there was strong positive staining for CK20, CK7 and very positive staining for Ki-67 in urothelial carcinoma in TUR resected tissues. Typically, the patterns of staining of urothelial tissue are positive for the both CK20 and CK7. In our case of immunohistochemical studies, both patterns of staining for villous adenoma and urothelial carcinoma were typical compared with past reports [5]. Ki-67 is associated with the growth fraction of a given human cell subset [26]. In Zarineh et al.’s case report of recurrent villous adenoma, the Ki-67 intensity was 20% [23]. The staining intensity of Ki-67 corresponded well with our result (<=30%). In contrast, the pattern of staining of urothelial carcinoma in our case was more than 70% positive for Ki-67. Bertz S et al. reported that Ki-67 and CK20 could be potential prognostic markers improving the risk stratification of pT1 bladder tumors [27]. The urothelial carcinoma of our case recurred within 3 months post surgery suggesting that a high expression of Ki-67 and CK20 may be associated with recurrence.

In this case, it was difficult to reveal the relationships between villous adenoma and urothelial carcinoma, because of the very small number of case reports. It is a distinct possibility that the two types of tumors could be incidental comorbid pathologies.

In the urinary tract, Powell et al. suggested that one case of villous adenoma progressed to villous adenocarcinoma [21]. No progressive cases of isolated villous adenomas have been found except for Powell’s report. Cheng et al. suggested that patients with isolated villous adenoma have an excellent prognosis on condition that complete resection is performed. However, the case with coexisting adenocarcinoma may have a good possibility of experiencing recurrence or distant metastasis, which will then require more aggressive treatments. Thus it is very important for us to avoid missing the presence of carcinomas which may coexist with villous adenoma and to follow up any malignant changes even in the isolated villous adenoma case. The number of current reports is insufficient to enable a valid prognosis.

## Conclusions

It was very difficult for us to distinguish between villous adenoma and urothelial carcinoma on ultrasonography, CT, MRI or cystoscopic examination. Therefore, pre-pathological villous adenoma of the bladder is extremely difficult to diagnose. Though, some case series of villous adenomas in the bladder were reported [5,24], this is only the second report of villous adenoma in the bladder of coexisting urothelial carcinoma that has been published in the literature. The most important point of this case is to avoid missing the presence of carcinomas which may coexist with villous adenoma and to follow up any malignant changes even in the isolated villous adenoma case.

## Consent

Written informed consent was obtained from the patient for publication of this manuscript and accompanying images. A copy of the written consent is available for review by the Editor-in-Chief of this journal.

## Abbreviations

VA: Villous adenoma; AD: Adenocarcinoma; UC: Urothelial carcinoma.

## Competing interests

The authors declare that they have no competing interests.

## Authors’ contributions

YK cared for the patient and drafted the report. SK cared for the patient. WO and TM revised and approved the final version of the manuscript. TS performed histopathological examinations. All authors reviewed the report and approved the final version of the manuscript.

## Pre-publication history

The pre-publication history for this paper can be accessed here:

http://www.biomedcentral.com/1471-2490/13/36/prepub
